# Yersinia enterocolitica in Crohn’s disease

**DOI:** 10.3389/fcimb.2023.1129996

**Published:** 2023-03-08

**Authors:** Xue Fang, Le Kang, Yi-Fan Qiu, Zhao-Shen Li, Yu Bai

**Affiliations:** Department of Gastroenterology, Changhai Hospital, Naval Medical University, Shanghai, China

**Keywords:** *Yersinia enterocolitica*, Crohn’s disease, immune system, inflammation bowel disease, gut microbiota

## Abstract

Increasing attention is being paid to the unique roles gut microbes play in both physiological and pathological processes. Crohn’s disease (CD) is a chronic, relapsing, inflammatory disease of the gastrointestinal tract with unknown etiology. Currently, gastrointestinal infection has been proposed as one initiating factor of CD. *Yersinia enterocolitica*, a zoonotic pathogen that exists widely in nature, is one of the most common bacteria causing acute infectious gastroenteritis, which displays clinical manifestations similar to CD. However, the specific role of *Y. enterocolitica* in CD is controversial. In this Review, we discuss the current knowledge on how *Y. enterocolitica* and derived microbial compounds may link to the pathogenesis of CD. We highlight examples of *Y. enterocolitica*-targeted interventions in the diagnosis and treatment of CD, and provide perspectives for future basic and translational investigations on this topic.

## Introduction

Crohn’s disease (CD), one of the main forms of inflammatory bowel disease, is characterized by patchy transmural inflammation that can involve any part of the digestive tract, from mouth to anus (Baumgart and Sandborn, 2012). Treatments for CD include the use of medication, alterations in diet and nutrition, and sometimes surgical procedures to repair or remove affected portions of digestive tract (Gajendran et al., 2018; Shi and Ng, 2018), however, no cure has been developed. In recent years, the global incidence of CD has been continuously increasing (Torres et al., 2017). Although a large number of studies have been conducted, the etiology of CD is still not clear. Many studies have revealed that there is a close relationship between the gut microbiota and CD. Dysbacteriosis is a typical symptom of CD patients, which includes reduced diversity, altered structural composition, and dysfunction of the gut microbiota (Gevers et al., 2014; De Cruz et al., 2015; Teh et al., 2021). The presence of gut microbes has been shown to be necessary for the occurrence of CD-related pathological changes in genetically susceptible mice (Kobayashi et al., 2014). Clinical observations from patients have demonstrated that CD lesions occur more frequently within the terminal ileum, which is an area with increased bacterial contact (Baumgart and Sandborn, 2012). Treatments, such as antibiotics and fecal microbiota transplantation, have been shown to alleviate CD, which also indicates the correlation between the intestinal microbiota and the inflammatory response (Selby et al., 2007). Genome-wide association studies have found more than 100 susceptibility loci in the genomes of CD patients (Sazonovs et al, 2022), and these susceptibility sites are mostly involved in microbial recognition and defense-related pathways (Hampe et al., 2001; Hampe et al., 2007). A nationwide case-control study showed that acute gastrointestinal infections increase the risk for CD, and speculated that the pathogens causing acute infectious gastroenteritis might play a role in the occurrence and development of CD (Axelrad et al., 2019).

*Yersinia enterocolitica* is the fourth most common bacterium causing acute infectious gastroenteritis in the European Union (EU) (Spackova et al., 2022). As a zoonotic pathogen which is widely distributed in nature, it is often transmitted to humans through consumption of contaminated food or water (Bottone, 1999). Compared with other bacteria, *Y. enterocolitica* is more frequently observed in CD lesions (e.g., CD specimens of mesenteric lymph nodes and Peyer’s plaques) (Lamps et al., 2003; Leu et al., 2013; Le Baut et al., 2018). Besides, there are many overlaps between *Y. enterocolitica* infection and CD in clinical symptoms and pathological manifestations, which often cause misdiagnosis and delays in treatment (Naddei et al., 2017; Cian et al., 2020). However, it is still unclear whether the presence of *Y. enterocolitica* in CD is a concomitant or accidental phenomenon or a contributing factor in the pathogenesis of CD. Therefore, we comprehensively investigate the relationship between *Y. enterocolitica* and CD from multiple aspects in this review, including pathological manifestations, diagnosis, treatment, epidemiology, and pathogenesis.

## Overview of Y. enterocolitica

*Y. enterocolitica* is a member of the phylum Proteobacteria, family Enterobacteriaceae, and genus *Yersinia*, which was first discovered and named in the mid-20th century (McIver and Pike, 1934; Schleifstein and Coleman, 1939; Hässig et al., 1949; Frederiksen, 1964). Generally, there are 18 classical strains (currently, more strains have been found using whole genome sequencing and gene alignment algorithms), most of which are regarded as environmental, non-virulent human pathogens (Sulakvelidze, 2000). Of the 18 strains, 3 are pathogenic to humans, namely, *Yersinia pestis* (the causative agent of plague, including the medieval ‘Black Death’), *Yersinia pseudotuberculosis*, and *Y. enterocolitica* (Achtman et al., 1999). The latter two can cause gastrointestinal disorders, of which, *Y. enterocolitica* is more relevant to gastrointestinal infections in humans. *Y. enterocolitica* can be further divided into a variety of biochemical and serological heterogeneous strains. To date, six biotypes (1A, 1B, 2, 3, 4, and 5) and more than 70 serotypes have been identified. Among them, biotype 1B strains (also named ‘New World’ or American strains due to their initial isolation in the USA) are considered highly virulent and lethal in mice, while biotype 1A strains are considered non-virulent due to the absence of the virulent plasmid pYV. Biotypes 2, 3, 4, and 5 (‘Old World’ or European strains) are low-virulence. The most common strains isolated from symptomatic humans belong to serobiotypes O: 3/4, O: (5 and 27)/(biotypes 2 and 3), O: 8/1B, and O: 9/2 (Lucero-Estrada et al., 2020).

**Figure 1:**
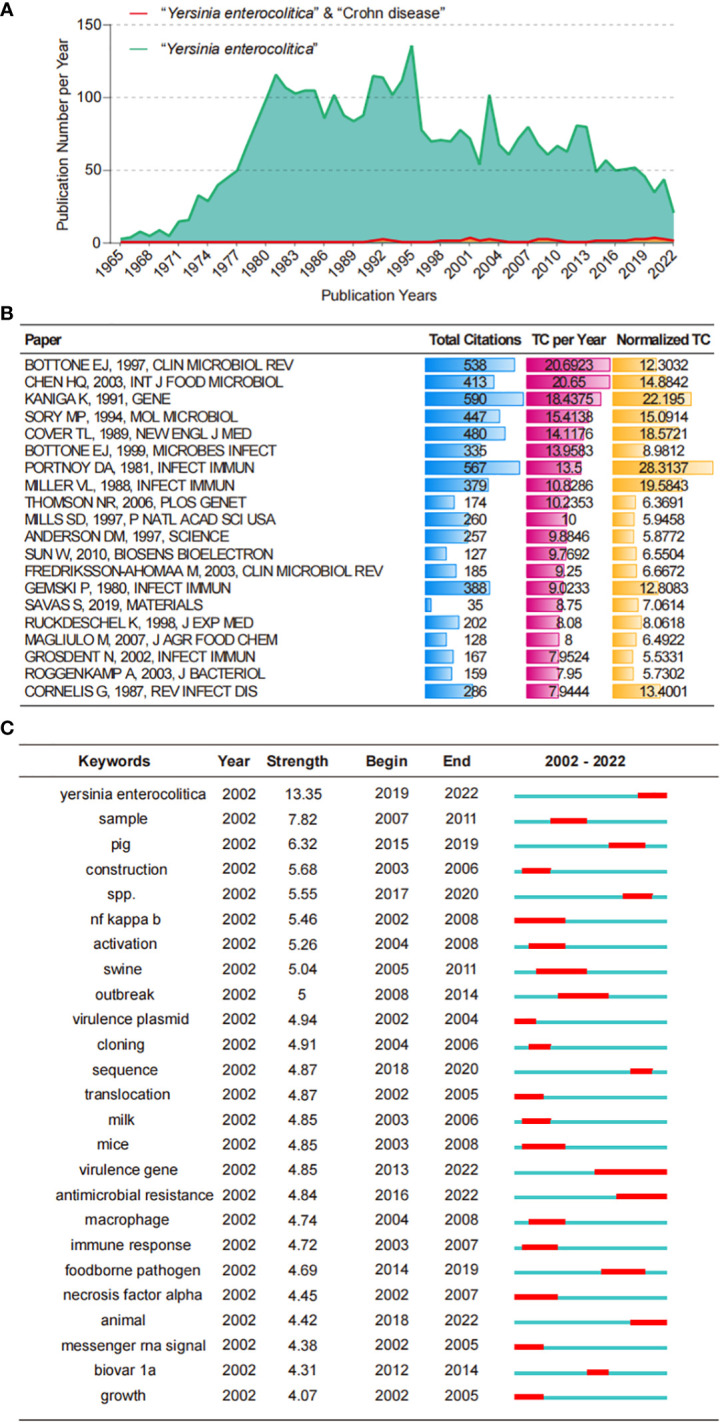
Bibliometric analysis of WoS core database output. **(A)** The growth productive trends of the topic “*Y. enterocolitica*” and “*Y. enterocolitica*” & “Crohn disease” research from 1900 to 2022. **(B)** Top 20 most cited papers in the topic “*Y. enterocolitica*” research from 1900 to 2022. **(C)** Top 25 Keywords with the Strongest Citation Bursts in the topic “*Y. enterocolitica*” research from 2002 to 2022. WoS, Web of Science.

**Figure 2:**
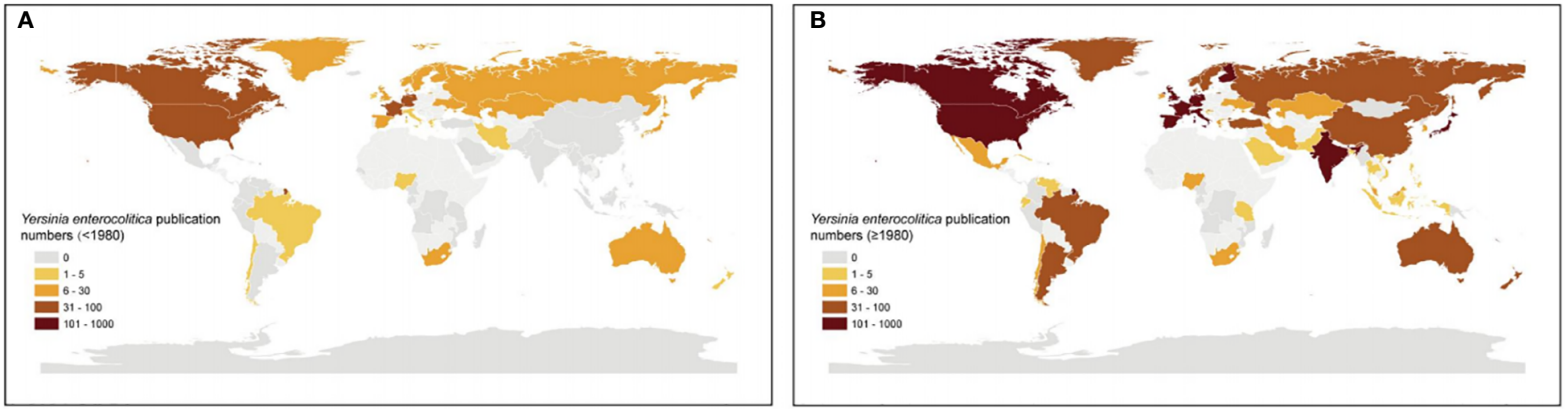
Worldwide *Y. enterocolitica* publication numbers by Bibliometric analysis. Worldwide *Y. enterocolitica* publication numbers for countries reporting data **(A)** before 1980 and **(B)** after 1980. Publication numbers were ranked into quintiles representing low (bright yellow) to intermediate (yellow) and high (brown).

A series of studies on *Y. enterocolitica* from 1930 to the present have focused on the identification and comparison of strains, the molecular mechanism of effector proteins in virulence and pathogenesis, clinical symptoms, and epidemiology (**Figures 1A–C**). Using scientometric analysis, we found that published studies regarding *Y. enterocolitica* were mainly from Europe and North America before 1980 (**Figure 2A**). After then, a number of studies were published from Asia, South America, and Africa (**Figure 2B**). In 1981, Kaneko et al. isolated *Y. enterocolitica* in Hokkaido, Japan, while retrospectively discovering that there had been several outbreaks of *Y. enterocolitica* community infection have occurred in Japan (Kaneko and Hashimoto, 1981). Sun et al. reported in the *Chinese Journal of Zoonoses* that 107 residents of Lanzhou City were infected after consuming contaminated beef in 1987, which was the earliest publication of an outbreak of *Y. enterocolitica* in China (Dianbin Sun and Wei, 1987). At present, the intimate relationship between *Y. enterocolitica* and CD has been widely recognized (Hugot et al., 2021). The epidemiology, clinical symptoms, pathological manifestations, and molecular mechanisms of this relationship are discussed in detail below.

## Are *Y. enterocolitica* and CD companions?

The epidemiology of *Y. enterocolitica* and CD seem to have a strong correlation. Compared to control groups, CD patients had a significantly higher prevalence of *Y. enterocolitica.* As many as 63% of patients with CD were found to have *Y. enterocolitica* based on the investigation by Kallinowski et al. (1998). As per Lamps et al., the detection rate of pathogenic *Y. enterocolitica* DNA in the bowel and mesenteric lymph nodes from patients with CD reached 31% (17/54), while all control tissues (40 cases of normal intestinal specimens, 30 cases of acute appendicitis, and 50 cases of active colitis) were negative (Lamps et al., 2003). Ahmad et al. showed that *Y. enterocolitica* was frequently present in patients with CD (7/69, 10.14%), and was significantly associated with the disease (p = 0.02) (Khan et al., 2021). Both *Y. enterocolitica* infection and the onset of CD in humans have obvious familial aggregation. One infection mode of *Y. enterocolitica* is a family-centered, small-scale outbreak (Black and Slome, 1988). Moreover, 12% to 18% of CD patients are reported to have at least one household member who suffers from the disease (Weterman and Pena, 1984). In a study on familial CD in Belgium, Herbert et al. found that CD patients and their relatives consumed more unpasteurized milk and cheese, undercooked beef, and pork than the control population (Van Kruiningen et al., 2005). It is speculated that these food with high chance of *Y. enterocolitica* contaminations may be a potential trigger of CD.

**Figure 3:**
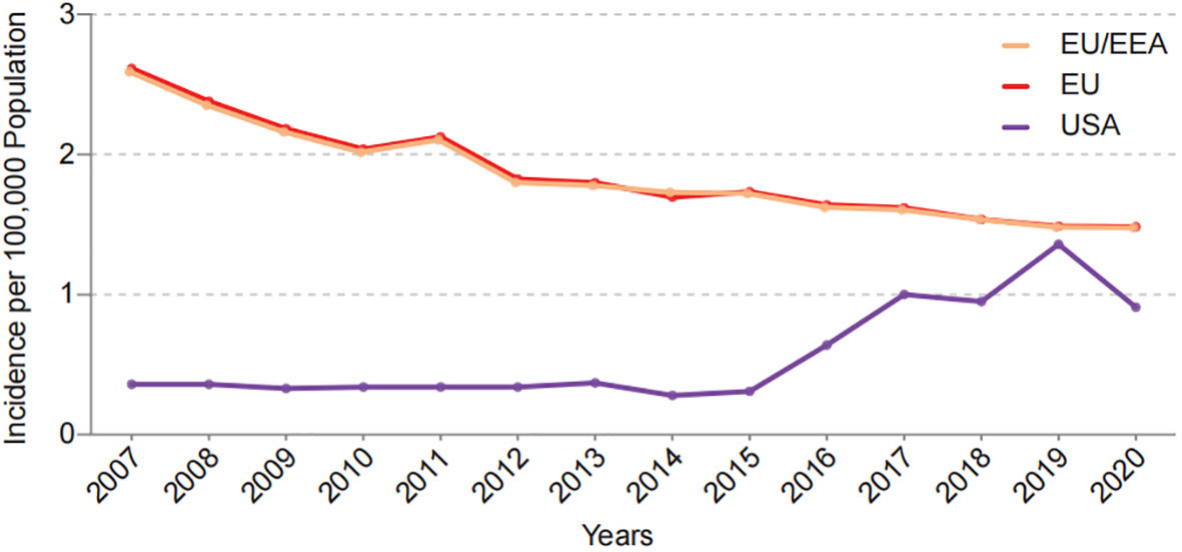
Incidence of Y.enterocolitica infections in EU/EEA, EU and USA per year from 2007 to 2020. Date come from European Centre for Disease Prevention and Control (ECDC) and Foodborne Diseases Active Surveillance Network (FoodNet).

Global trends in the prevalence of CD and *Y. enterocolitica* infection are also similar. Historically, the outbreaks and epidemics of *Y. enterocolitica* infection and CD cases mostly have been reported in the northern hemisphere such as North America and Northern Europe (Bottone, 1977; Molodecky et al., 2012). Currently, there has been a weakened north-south gradient in *Y. enterocolitica* transmission and CD in recent decades, which may be attributed to the advancement of global modernization. According to data from the European Centre for Disease Prevention and Control (ECDC) and Foodborne Diseases Active Surveillance Network (FoodNet) in the United States, the incidence of *Y. enterocolitica* in Europe and the United States has decreased over the past decade (**Figure 3**). Meanwhile, an increasing incidence of *Y. enterocolitica* has been found in some low-risk areas in the past. Meta-analysis of the global annual incidence of *Y. enterocolitica* in gastroenteritis cases between January 1, 2000 and December 31, 2019 showed that Africa and the Eastern Mediterranean rank first and second respectively in the global prevalence of *Y. enterocolitica* (Riahi et al., 2021). Correspondingly, newly industrialized regions from Asia, Africa, and South America are experiencing an increase in CD cases (Giegerich et al., 2018), and New Zealand and Australia are among the top four countries with the highest annual incidence of CD at present (Torres et al., 2017). Notably, bacterial infections such as Yersinia are more likely to be diagnosed and treated than those of a chronic disease such as CD, which may also contribute to global incidence changes. Taken together, these evidences suggest that *Y. enterocolitica* has a strong epidemiological relationship with CD. However, the mechanism underlying this association is unknown. Furthermore, it is unclear if interactions between *Y. enterocolitica* and CD-susceptible individuals initiate the onset of disease. Thus, further investigations regarding *Y. enterocolitica* infection and CD patients are needed.

## Whether *Y. enterocolitica* and CD share pathogenesis?

**Figure 4:**
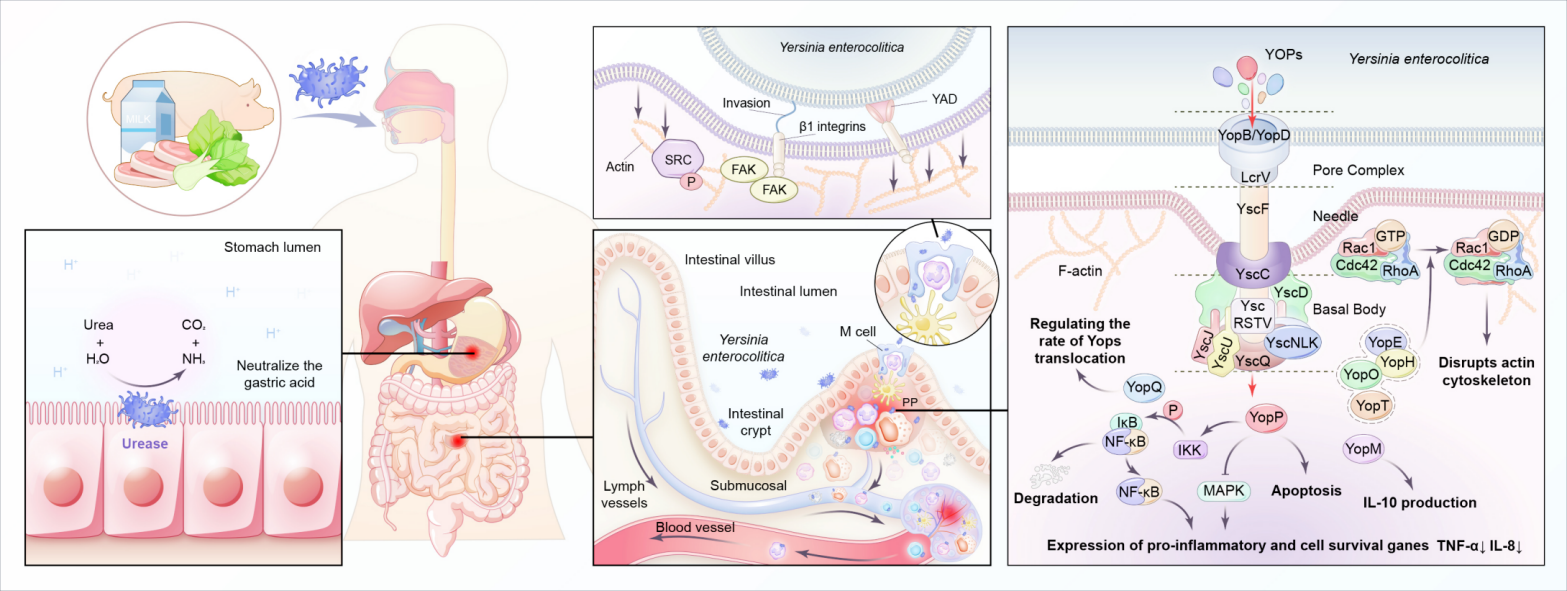
Pathogenic mechanisms of Y. enterocolitica.

Infection of *Y. enterocolitica* disturbs immune balance in the host. The pathogenesis depends on multiple chromosome-encoded and plasmid-encoded virulence determinants (**Table 1**) (**Figure 4**). Once entering the digestive tract, *Y. enterocolitica* first passes through the gastric acid barrier with the help of urease (Righetto et al., 2020). After reaching the distal small intestine, it preferentially adheres to and invades M cells in the follicular-associated epithelium (FAE). The interactions between bacterial adhesins and β1-integrins on the surface of M cells induce *Y. enterocolitica* internalization by stimulating the remodeling of actin filaments and forming a polymerized actin vacuole (Schulte et al., 2000). Then the vacuole transports from the apical to the basal side of the M cell, where *Y. enterocolitica* is expelled into the lymphoid follicles of the Peyer’s patches (Autenrieth and Firsching, 1996). With the help of Yad and invasion proteins, *Y. enterocolitica* adheres to the host immune cells (including macrophages, neutrophils, dendritic cells, and monocytes) surfaces (El Tahir and Skurnik, 2001; Heise and Dersch, 2006). At the same time, the T3SS injection system forms a channel between *Y. enterocolitica* and the target cell and injects cytotoxic effector *Yersinia* outer-membrane proteins (Yops) into the cytoplasm of the host cell, enabling the bacteria to evade the host immune system (Cornelis, 2006; Cornelis, 2010). For example, YOPs, such as YopE, YopH, YopT, and YopO, inhibit the phagocytosis of macrophages and leukocytes by disrupting the actin cytoskeleton. YopP inhibits multiple signaling pathways, including TNF-α and IL-8, and exploits lipopolysaccharide signaling to trigger apoptosis in infected macrophages and dendritic cells (Denecker et al., 2002). In addition, the immunomodulatory protein Gal-1 can promote the replication of *Y. enterocolitica* in PP, and this protein has been shown to play a role in limiting bacterial clearance (Davicino et al., 2017). After the incubation period of 4–7 days, the engulfed bacteria migrate to the mesenteric lymph nodes, where they multiply and cause abscess formation (Hein et al., 2000). Systemic infection occurs with the migration of *Y. enterocolitica* along with the lymphatic fluid or blood and may cause extraintestinal complications, such as sepsis (Lai et al., 2014), deep organ abscess (Viteri et al., 1981; Demes et al., 2021), and arthritis (Appel et al., 2002). Moreover, YadA-mediated complement evasion may result in persistent pathogen presence (China et al., 1993).

**Table 1:** Comparative analysis of virulence factors in *Y. enterocolitica* and their potential effect in Crohn’s disease.

Classification	Effector	Genomic location	Biological Activity	Target	Effect/Feature	Report in Crohn’s disease
**Attachment and invasion**	**yadA**	**Plasmid**	**Fibrous, lollipop-like structures**	**Hydroxyproline rich peptides collagen I, II, IV, and laminin MAPK**	**Mediates cell adhesion and host cell responses induction, like cytokine production, autoagglutination, and serum resistance** (El Tahir and Skurnik, 2001; Muhlenkamp et al., 2017).	
	**invA**	**Chromosomal**		**β1 integrins**	**Mediates cell adhesion, effective bacteria translocation into M cells and Peyer’s patches colonization** (Schmid et al., 2004).	
	**ail**	**Chromosomal**	**The C-terminal half of loop 2**	**Factor H**	**Cooperates with YadA to ensure a high level of serum resistance to *Y. enterocolitica*** (Felek and Krukonis, 2009; Dhar and Virdi, 2014).	
**Secretion system**	**ysc T3SS**	**Plasmid**	**LcrV, YopB,YopD and Ysc family**	**Cytoplasmic membrane**	**Forms a multimeric integral membrane complex in the membrane of eukaryotic cells to help Yops translocation** (Montagner et al., 2011).	**Treatment of mice with rLcrV leads to suppression of TNFα and IFNγ *via* amplification of IL-10 and inhibition of neutrophil chemotaxis. (Correlation)** (Motin et al., 1994)
	**ysa T3SS**	**Chromosomal (highly virulent Y. enterocolitica O:8/0B strains)**	**YspA,YspE, YspF, YspI, YspK, YspL, YspM and YspP**	**Cytoplasmic membrane**	**Control of Yops secretion which is important for early infection and invasion of the epithelial M-cells in the Peyer’s patches** (Young et al., 1999; Marenne et al., 2003; Venecia and Young, 2005).	
	**yts1 T2SS**	**Chromosomal(Y. enterocolitica subsp. enterocolitica)**	**yts1, yts2**	**Interaction of free-living bacteria with their environment**	**Helps the bacteria survived in an environmental habitat. Yts1 has been shown to be involved in dissemination and colonization of deeper tissues, like liver and spleen** (Iwobi et al., 2003; Shutinoski et al., 2010).	
	**yts2 T2SS**	**Chromosomal**				
**Yop Effectors**	**YopE**	**Plasmid**	**GAP**	**RhoA, Rac1 and Cdc42**	**Actin depolymerization, inhibition of phagocytosis, of reactive oxygen species ROS production, and of the inflammatory response** (Aepfelbacher, 2004; Schotte et al., 2004).	**YopE not only attenuates the production of IL-8 (CXCL8), an important chemoattractant and activator for neutrophils, but also affects neutrophil migration, which is largely dependent on Rho-mediated signaling** (Viboud et al., 2006; Amulic et al., 2012; Grabowski et al., 2017).
	**YopT**	**Plasmid**	**Cysteine protease**	**Releasing of Rac, RhoA, Cdc42 from the membrane and thus leading to their inactivation**	**Actin depolymerization, inhibition of phagocytosis** (Iriarte and Cornelis, 1998; Viboud et al., 2006).	**Similar phenotype as YopE.**
	**YopO**	**Plasmid**	**N-terminal serine/threonine kinase domain, a C-terminal**	**Rac and RhoA**	**Actin depolymerization** (Park et al., 2007).	**YopO has a guanine nucleotide dissociation inhibitor (GDI) domain, which binds to GDP-bound forms of Rac and RhoA, and thus prevents their activation, resulting in the inhibition of stress fibre formation, impairs the phagocytosis of bacteria by macrophage** (Barz et al., 2000).
			**GDI domain and an actin binding domain**			
	**YopH**	**Plasmid**	**PTPase**	**P130Cas, FAK, Paxillin, Fyb, SKAPHOM, p85, Lck**	**Disruption of peripheral focal complexes, impairing of T and B cell activation, inhibition of phagocytosis, of ROS production, and of the inflammatory response** (Guan and Dixon, 1990; Bliska et al., 1991).	
	**YopP**	**Plasmid**	**Cysteine protease**	**MAKKs, IKKβ, TRAF2, TRAF6, IKKα, IKKβ, and IκBα**	**Prevents NF-κB and MAPK activation, inhibition of inflammatory response, apoptosis in macrophages and dendritic cells** (Mills et al., 1997; Boland and Cornelis, 1998; Ruckdeschel et al., 2001).	**YopP makes the down-regulation of cytokines, chemokynes and adhesion molecules** (Boland and Cornelis, 1998)**, and inhibits the recruitment and activation of macrophages and natural killer cells to the site of infection, so helps the bacteria evade the host inflammatory response** (Navarro et al., 2005).
	**YopM**	**Plasmid**	**LRR domain**	**Alpha-thrombin, PRK2, RSK1, caspase-1**	**Disruption of inflammasome formation and caspase-1 activation, depletion of NK cells, induction of IL-10 production** (Kobe and Deisenhofer, 1994; Kerschen et al., 2004; McPhee et al., 2010; LaRock and Cookson, 2012).	**YopM might interact physically with CARD15 to induce an inhibition of the NF-κB by an alternative pathway, for both of them contain the LRR domain** (Hugot et al., 2003).
	**YopQ**	**Plasmid**	**Not known**	**Inflammasome**	**Regulation of the translocation rate of Yop effectors into eukaryotic cells, and prevention of inflammasome activation by inhibiting detection of the T3SS by the innate immune system and/or by regulating the rate of Yops translocation** (Brodsky et al., 2010).	
**Enterotoxin**	**YstA,YstB,YstC**	**Chromosomal**	**The C-terminal 13 amino acid regions**	**GC-C/cGMP**	**Accumulation of fluids in the intestinal lumen, which leads to diarrhea** (Delor et al., 1990; Revell and Miller, 2001).	
**Urease**	**Urease**	**Chromosomal**		**Catalysing the hydrolysis of urea to ammonia and carbon dioxide**	**Helps Yersiniae to live as saprophytes in the environment, protect *Y. enterocolitica* during its passage through the stomach** (de Koning-Ward et al., 1994).	
**Yersiniabactin**		**Chromosomal(HPI)(highly virulent Y. enterocolitica subsp. enterocolitica)**			**Regulating iron uptake witch strongly correlates with Y. enterocolitica ability to proliferate and with mouse virulence** (Pelludat et al., 1998).	
**endotoxin**	**LPS**	**Chromosomal**	**lipid A (or endotoxin)**	**TLR4, CD14**	**A powerful activator of the host immune system, stimulating leukocyte inflammatory cytokine production, high concentrations of LPS cause cytokine overproduction-mediated sepsis** (Erridge et al., 2002; Maldonado et al., 2016)	

invA, invasin A; ail, attachment invasion locus; CARD15, caspase activation and recruitment domain 15; FAK, focal adhesion kinase; Fyb, Fyn-binding protein; GAP, GTPase-activating protein domain; GDI, guanine nucleotide dissociation inhibitor; HPI, high-pathogenicity island; IKK, inhibitor-kappa B kinase; LPS, lipopolysaccharide; LRR, leucine-rich repeats; MAKK, mitogen-activated kinase kinase; MAPK, mitogen-activated protein kinase; P130Cas, p130 Crk-associated substrate; PRK2, protein kinase C-like 2; PTPase, protein tyrosine phosphatase; ROS, reactive oxygen species; RSK1, ribosomal S6 protein kinase 1; T2SS, type II secretion system; T3SS, type III secretion system; TLR4, Toll-like receptor; Yops, Yersinia outer-membrane proteins; Ysa, Yersinia secretion apparatus; Ysc, Yop secretion; Ysp, Yersinia secreted protein; Yst, Yersinia heat-stable toxin.

Both *Y. enterocolitica* infection and CD exhibit immune defects during pathogenesis, with some immune responses shared. A break of the balance between immunosurveillance and immune escape of the intestinal microbiota is crucial in the pathogenesis of CD. For example, both mutations in MUC2, which encodes for intestinal mucus, and the emulsifiers commonly found in Western diets are risk factors for CD, as they both lead to the destruction of the mucus layer and create opportunities for the translocation of intestinal pathogens, such as *Y. enterocolitica* (Boltin et al., 2013; Chassaing et al., 2015). The susceptibility genes of CD are mostly related to microbial recognition and defense. CARD15, originally named NOD2, is the first identified susceptibility gene of CD. Its encoding protein NOD-like receptors (NLRs) expressed in the cytoplasm of many innate immune cells, respectively, is a main group of pattern recognition receptors (PRRs) responsible for recognizing intestinal microbiota (Al Nabhani et al., 2017). Individuals carrying CARD15 mutations were reported to present with abnormal immune responses to *Y. enterocolitica* infection and are subsequently diagnosed with CD (Safa et al., 2008). Toll-like receptors (TLR) expressed in the membrane of many innate immune cells, is another main group of PRRs. TLR1^-/-^ mice with acute *Y. enterocolitica* infection exhibit CD-like symptoms post-infection, including poor weight gain, dysbiosis, long-term chronic inflammation, and an increased anti-commensal immunity as compared to their wild-type (WT) littermate control mice (Kamdar et al., 2016). The autophagy process, which is also affected by *Y. enterocolitica* infection, has a vital role in downstream antibacterial mechanisms mediated by the TLR and NLR signaling pathways. Murthy et al. found that *Y. enterocolitica* could activate caspase 3 after infection, leading to the accelerated degradation of the CD-susceptible autophagy gene ATG16L1 (T316A), which in turn, led to reduced autophagy, impaired pathogen clearance of ileal *Y. enterocolitica*, and increased secretion of TNF-α and IL-1β (Murthy et al., 2014). The detailed molecular mechanisms involved are the effects of *Y. enterocolitica* and derived microbial compounds on immune cells. For example, YopE has a Rho GTPase-activating protein domain (GAP), which targets RhoA, Rac1 and Cdc42. By hitting Rho-GTPases, YopE not only attenuates the production of IL-8 (CXCL8), an important chemoattractant and activator for neutrophils, but also affects neutrophil migration, which is largely dependent on Rho-mediated signaling (Viboud et al., 2006; Amulic et al., 2012; Grabowski et al., 2017). YopO has a guanine nucleotide dissociation inhibitor (GDI) domain, which binds to GDP-bound forms of Rac and RhoA, and thus prevents their activation, resulting in the inhibition of stress fiber formation, impairs the phagocytosis of bacteria by macrophage (Barz et al., 2000). YopP inhibits multiple signaling pathways, including the NF-κB and MAPKs pathways, and exploits lipopolysaccharide -related signaling pathway to trigger apoptosis in infected macrophages and DC (Ruckdeschel et al., 2001). YopP makes the down-regulation of cytokines, chemokynes and adhesion molecules (Boland and Cornelis, 1998), and inhibits the recruitment and activation of macrophages and natural killer cells to the site of infection, so helps the bacteria evade the host inflammatory response (Navarro et al., 2005).

*Y. enterocolitica* infection can evoke a long-term immune response and gut microbiota alteration, then lead to developing of CD in genetically susceptible individuals. As Netea et al. reported, the defects in the recognition pathways of TLR5 and NOD2 led to a defective inflammatory response to *Y. enterocolitica* and long-term abdominal inflammation (Netea et al., 2010). Denise et al. suggested that constant disruption in the communication between the immune system and tissue systems following clearance of an acute infection denotes a turning point in which both tissue and immune homeostasis were altered *via* long-term reprogramming (Fonseca et al., 2015). Therefore, we suggested that long-term reprogramming of immune cells after *Y. enterocolitica* infection, as well as profound and persistent remodeling of MAT and MLN, leads to the development of chronic inflammatory conditions through a phenomenon known as immunological scarring. Of course, this speculation needs to be confirmed by further studies.

## *Y. enterocolitica* in the diagnosis of CD

It was noted decades ago that there were many similar pathological manifestations and clinical symptoms between CD and *Y. enterocolitica* infection (**Table 2**). For instance, the location of lesions in both diseases is most frequently presented in the terminal ileum - areas with increased bacterial contact (Payne et al., 1987). Specifically, the Peyer’s patches and isolated lymphoid follicles in the small intestine, where *Y. enterocolitica* primarily colonizes (Revell and Miller, 2001), are also sites for typical aphthoid lesions during the early stage of CD (Lockhart-Mummery and Morson, 1960; Fujimura et al., 1996; Krauss et al., 2012). Different from the ulcers caused by local intestinal ischemia, Behcet’s disease and intestinal tuberculosis tend to occur on the mesenteric side of the intestinal lumen (Sunada et al., 2009). The main clinical symptoms in humans infected with *Y. enterocolitica* include abdominal pain, diarrhea, and vomiting. Endoscopic observation shows ileal edema, thickening of the intestinal mucosal, epithelioid granuloma, lymphoid tissue swelling, and an increased number of transmural blood vessels. Microscopic observation shows fossa, crypt abscess, and plasma cell and lymphocyte infiltration. All of which are consistent with the clinical and histological manifestations of CD (Gleason and Patterson, 1982; Bronner, 2004; Franczak et al., 2018). Creeping fat, an extra-intestinal manifestation of CD, is manifested by the migration and wrapping of mesenteric adipose tissue (MAT) to around sites of intestinal inflammation. Creeping fat is visually striking alongside the patchy lesions (Sheehan et al., 1992; Peyrin-Biroulet et al., 2007). Connie et al. showed that translocation of the gut microbiota is the main driving factor leading to the formation of creeping fat (Ha et al., 2020). Interestingly, the remodeling of MAT was also found in *Y. enterocolitica*-infected mice (Antonopoulos et al., 2008; Han et al., 2017).

**Table 2:** Clinical symptoms and pathological manifestations of CD and *Y. enterocolitica* infection.

	Yersinia enterocolitica infection	Crohn’s diseases
Clinical symptoms	Abdominal pain, distention, diarrhea, and fever	Abdominal pain, distention, diarrhea, and fever
Clinical course	Acute or subacute (days to weeks), chronic(months or even years)	Lifetime with remission and exacerbation
Etiologies	Yersinia enterocolitica infection	Multifactors involving immune, genetic and environmental factors
Onset location	It occurs mostly in the terminal ileum	Can involve any part of the digestive tract, from mouth to anus, usually in the terminal ileum
Endoscopic appearance	Ileal edema, thickening of the intestinal mucosal, epithelioid granuloma, lymphoid tissue swelling, and skip circular/elliptical ulcers along with Peyer’s patches	Ileal edema, thickening of the intestinal mucosal, epithelioid granuloma, lymphoid tissue swelling, skip circular/elliptical ulcers along with Peyer’s patches, pebble sign; longitudinal ulcers, strictures, and fistulas (late CD)
Histopathology appearance	Neutrophilic and lymphocytic infiltrates as well as cryptitis, epitheloid granuloma and crypt abscesses	Neutrophilic and lymphocytic infiltrates, cryptitis, epitheloid granuloma, crypt abscesses and nerve hyperplasia

In current clinical practice, the diagnosis of CD always needs to determine whether *Y. enterocolitica* infection exists. While positive cultures obtained from the mesenteric lymph nodes, pharyngeal exudates, peritoneal fluid, or blood can successfully diagnose *Y. enterocolitica* infection (Centers for Disease, 2011), this laborious and time-consuming method has been gradually replaced by molecular detection, including serological testing, such ELISA (Laporte et al., 2015) and agglutination (Paerregaard et al., 1991; Sonnevend et al., 2005) detection of the presence of specific antibodies (such as IgG, IgG, IgA, and IgM) against YOPs (such as LorV (V antigen), YopD, and Yop M) produced by B cells with the help of intestinal dendritic cells. Triantafillidis et al. recommended that all patients with terminal-ileitis as evidenced by endoscopic and histological images should be tested for YOP-specific antibodies to determine whether the terminal-ileal CD is associated with *Y. enterocolitica* infection (Triantafillidis et al., 2020). However, serological testing also has many concomitant difficulties, including cross-reactions with other intestinal microbes (Golkocheva-Markova et al., 2008). Currently, more sensitive, highly accurate and precise methods, such as PCR and multiplex PCR, have been used to clinically and preclinically detect the transposon and virulence genes of pathogens within mucosal tissues (Zakharchuk, 1965; Ye et al., 2014; Engberg et al., 2021). High-throughput sequencing technologies, such as metagenomic sequencing (Kim et al., 2021), will help to increase our understanding of the diversity and function of the gut microbiota and promote the development of new molecular diagnostic tools, thereby achieving accurate diagnosis and treatment of diseases.

## *Y. enterocolitica* in the treatment of CD

*Y. enterocolitica* infection should be considered in the treatment of CD. Although *Y. enterocolitica* infection is self-limiting in most cases, there is a risk of systemic infection if patients receive immunosuppressive therapy due to presumptive CD. Timely antibiotic treatment is necessary for some patients with *Y. enterocolitica* infection complications. A study by George et al (Arnold et al., 2002). showed that ciprofloxacin, a first-line anti-*Y. enterocolitica* antibiotic may be an effective agent when added to the treatment of moderately active, resistant CD. We speculated that this result might be related to the clearance effect of ciprofloxacin on *Y. enterocolitica*. In addition, the presence of the high-pathogenicity island (HPI) in highly virulent strains (e.g., type 1B/O:8), which is responsible for the siderophore yersiniabactin-mediated iron uptake, facilitates the absorption and utilization of iron by *Y. enterocolitica* and promotes its growth under iron-limiting conditions (Bancerz-Kisiel et al., 2018). Thus, although iron-deficiency anemia is a common extraintestinal complication of CD (Stein et al., 2010; Azghari et al., 2016), *Y. enterocolitica* infection should be excluded prior to administering iron supplementation to anemic CD patients, as increased iron levels may increase the risk for sepsis (Cross et al., 2015). In addition, *Y. enterocolitica* should be considered in the treatment of CD, for it may alter drug activity through metabolism, thereby enhancing or inhibiting the clinical effect of the treatment.

Eliminating *Y. enterocolitica* may improve the symptoms of CD patients. For example, exclusive enteral nutrition (EEN) is used as an effective first-line treatment for inducing remission in pediatric Crohn’s disease (Ruemmele et al., 2014; Levine et al., 2019). It consists only of liquid nutrition that eliminates suspected food causative agents such as allergenic proteins, refined sugar, and pathogenic microorganisms including the Y. enterocolitica. In addition, there is evidence that EEN modulates the activity and structure of intestinal microbiota and consequently attenuates inflammation (Lionetti et al., 2005). The treatment of CD with probiotics has also gained substantial research interest (Guslandi et al., 2000; Sokol et al., 2008), as they exhibit high safety. Probiotics also have inhibitory effects on the presence of *Y. enterocolitica* infection through several mechanisms: 1) production of inhibitory substances, 2) blockade of intestinal surface adhesion sites, 3) nutrient competition, and 4) stimulation of mucosal and systemic immunity. For example, Bujalance et al. found that 20 strains of lactic acid bacteria had inhibitory effects on *Y. enterocolitica* and this inhibition was mainly attributed to the decrease in pH caused by glucose fermentation by lactic acid bacteria (Bujalance et al., 2014). *Lactobacillus fermentum* attenuates the proinflammatory effect of *Y. enterocolitica* on human epithelial cells (Frick et al., 2007). Both *in vivo* and *in vitro* experiments have shown that the probiotic *Escherichia coli* strain Nissle 1917 inhibits the invasion of *Y. enterocolitica* by secreting antibacterial compounds (Altenhoefer et al., 2004). Within the patient‐based studies, administration of *Saccharomyces boulardii* has been reported helpful in maintaining remission and bowel sealing (Garcia Vilela et al., 2008). However, we do note that, in some studies, probiotics did not perform better than placebo in inducing the remission of CD (Limketkai et al., 2020). More researches are needed to determine what strains and at what dose probiotics become more useful as part of a clinical intervention.

As described above, some strategies targeting *Y. enterocolitica* may have unexpected potential diagnostic and therapeutic applications in the treatment of CD. Development of agents with specific toxicity for *Y. enterocolitica* or its virulent strains is promising and merits more investment. In addition, based on the immunosuppressive effect of some Yops, these proteins may become innovative biological agents for the treatment of CD by inhibiting the release of chemokines and inflammatory factors and affecting the migration of neutrophils (Grabowski et al., 2017), thereby alleviating CD inflammation. The establishment of engineered bacteria or vaccines of *Y. enterocolitica* for reliable targeted drug delivery and intestinal immune regulation may provide unexpected potential treatments for abnormal submucosal immune responses in CD.

## Discussion

In the past decades, there has been a substantial increase in the understanding of microbiota-host interactions. Specifically, there has been considerable advancements regarding the molecular mechanisms of *Y. enterocolitica* in host immune disorders. However, the role of *Y. enterocolitica* in CD pathogenesis remains unclear for several reasons.

First, some studies have declared that they failed to find differences in the *Y. enterocolitica* infection rates between CD patients and controls (Hugot et al., 2021). One reason for this different conclusion may be the detection method. Some clinical analyses (16S rRNA and metagenomic sequencing) of the gut microbiota in CD patients have primarily focused on stool samples (Khan et al., 2021). Using only stool to assess the gut microbiota can potentially dilute the signal of low-abundance bacteria, such as *Y. enterocolitica*, which are typically diluted by the high abundance of “transient bacteria”. Therefore, more appropriate clinical samples, such as mucosal lesions, Peyer’s patches, mesenteric lymph nodes, and creeping fat should be detected. Another factor that hinders understanding the close association between *Y. enterocolitica* and CD is the complex and diverse clinical manifestations of *Y. enterocolitica* infection. The clinical pathogenicity of different serotypes of Y. enterocolitica is different, and similarities of *Y. enterocolitica* infection with CD may only manifest with highly virulent strains. Thus, focusing on the role of pathogenic *Y. enterocolitica* in CD is essential to furthering this area of research. Additionally, other pathogens are also involved in the pathology of CD, such as adherent-invasive *Escherichia coli*, as reviewed by Rolhion et al (Rolhion and Darfeuille-Michaud, 2007). and Palmel et al (Palmela et al., 2018).

Second, the function and mechanism of Y. enterocolitica in the pathogenesis of CD are still uncertain. It has been reported that some *Y. enterocolitica*-positive patients were subsequently diagnosed with CD (Lamps et al., 2001; Zippi et al., 2006). However, evidence of an association between *Y. enterocolitica* and CD does not directly indicate a causal relationship between the two, nor does it rule out the possibility that *Y. enterocolitica* is only a confounding factor in the etiology of CD. In addition, CD patients are often immunodeficient, and as such, receive immunosuppressive treatment, which leads to an increased risk of infection with pathogenic bacteria, such as *Y. enterocolitica*. However, whether secondary infection of *Y. enterocolitica* affects the incidence rate, severity, and recurrence rate of CD is also worthy of further study.

Specifically, a multicenter, prospective study with large sample size, as well as additional studies using relevant genetically susceptible and gnotobiotic mice, coculture of gut-microbiota, immune cells and intestinal organoid on a chip, advanced sequencing technologies such as single-cell transcriptome and spatial transcriptome are needed to elucidate the development and progression of *Y. enterocolitica* in CD. Understanding the molecular mechanisms of the pathogenesis of these two diseases provides prospects for better diagnosis and treatment. More rapid and sensitive tools for the detection of *Y. enterocolitica* and strict food monitoring and management are conducive to reducing the chance of pathogenic *Y. enterocolitica* infection. They will help control gastrointestinal infections and even possibly CD. Innovative biological agents based on *Y. enterocolitica*, such as YOPs, genetically engineered bacteria, and vaccines, may also have unexpected potential therapeutic applications in the treatment of CD. In addition, considering the infection of *Y. enterocolitica* in CD patients and taking more proper management is very important. In summary, studying the relationship between *Y. enterocolitica* and CD may provide a basis for clarifying the etiology and pathogenesis of CD, formulating a reasonable and practical treatment plan, and determining the prognosis of the disease.

## Author contributions

YB and Z-SL conceived the original idea. XF, LK, and Y-FQ collected and wrote the manuscript with contributions from all other authors. All authors contributed to the article and approved the submitted version.
